# Using a Weekly Patient-Reported Outcome Questionnaire to Track Acute Toxicity in Patients Undergoing Pelvic Radiotherapy for Gynecologic Cancers

**DOI:** 10.3390/curroncol29050270

**Published:** 2022-05-05

**Authors:** Matthew Chan, Robert Olson, Vincent Lapointe, Jeremy Hamm, Francois Bachand, Caroline Holloway, Christina Parsons, Peter Lim

**Affiliations:** 1 BC Cancer–Vancouver, Vancouver, BC V6T 1Z4, Canada; matthew.chan@bccancer.bc.ca (M.C.); vlapoint@bccancer.bc.ca (V.L.); jhamm@bccancer.bc.ca (J.H.); cparsons@bccancer.bc.ca (C.P.); 2 Department of Surgery, Faculty of Medicine, University of British Columbia, Vancouver, BC V6T 1Z4, Canada; rolson2@bccancer.bc.ca (R.O.); fbachand@bccancer.bc.ca (F.B.); cholloway@bccancer.bc.ca (C.H.); 3 BC Cancer–Prince George, Prince George, BC V2M 7E9, Canada; 4 BC Cancer–Kelowna, Kelowna, BC V1Y 5L3, Canada; 5 BC Cancer–Victoria, Victoria, BC V8R 6V5, Canada

**Keywords:** patient reported outcomes, acute toxicity, pelvic radiotherapy, gynecologic cancers

## Abstract

There are limited patient-reported outcome (PRO) data tracking changes in toxicity in patients actively undergoing radiotherapy. Between 2015–2019, acute toxicity was prospectively measured in 698 patients undergoing a 5-week course of pelvic radiotherapy for gynecologic cancers using a weekly PRO questionnaire. Our questionnaire was able detect a pattern of onset and resolution of acute gastrointestinal (GI) and genitourinary (GU) toxicity in 27 out of 32 questions. Logistic regression analysis showed that increasing GI and GU toxicity at week 2 could predict for severe toxicity at week 5. However, due to a low number of severe events, univariate results could not be productively added to a multivariate model. We observed a >70% response rate for all sections of the questionnaire, except for questions on sexual and vaginal health, which had a 13% average response rate. By demonstrating that PRO data can be used to track acute toxicity during radiotherapy, there is a need to further examine how this tool may be implemented in the clinic to provide complex, adaptive care, such as early side effect management, and modifying radiation delivery in real-time.

## 1. Introduction

The use of patient-reported outcome data (PRO) in the clinic has been shown to improve symptom detection, clinical management, and outcomes in oncology (e.g., symptom control, health-related quality of life) [1,2]. A recent randomized trial with patients undergoing chemotherapy for metastatic cancers has further suggested a possible survival benefit with PRO monitoring [3].

Comparatively, there have been fewer studies assessing the utilization and benefit of PROs in patients undergoing radiotherapy, despite often having extended treatment courses, and in contrast, being used more often as a primary modality with curative intent. The latter emphasizes the importance of utilizing PROs to reduce treatment interruptions and improve radiotherapy completion rates. PROs may aid in establishing thresholds at which to implement early supportive care measures, which may reduce downstream severe toxicity. There is also the potential to evaluate toxicity when introducing treatment modifications, such as changes in radiation dose-fractionation, or the inclusion of additional lymph node regions in treatment volumes. 

In April 2015, six regional cancer centres across British Columbia began collecting PROs in patients undergoing radiotherapy for gynecologic cancers. This was conducted using a questionnaire composed of five validated questionnaire subsets focusing on bowel function, urinary function, abdominal problems, gynecological issues, and general health. While the feasibility of its implementation had been previously described, we sought to summarize and evaluate the initial patient responses with a particular focus on the ability of our questionnaire to track acute toxicity during radiotherapy [4]. 

## 2. Materials and Methods

We retrospectively evaluated PRO questionnaire responses from patients diagnosed with gynecologic cancers undergoing at least a 5-week course of pelvic radiotherapy at one of our six regional BC Cancer centres between April 2015 and May 2019. The survey was comprised of 49 questions derived from 5 independently validated questionnaire subsets [4]. These included EPIC Bowel/Urinary 2.0, PRO-CTCAE GI, EORTC QLQ CX24, and EuroQol EQ-5D-5L (questionnaire is shown in Supplementary Figure S1). The questionnaire was administered on a tablet device prior to first treatment (either at the time of CT simulation or new patient education session), 1–5 times at weekly intervals during radiotherapy (depending on the treatment centre), and at each subsequent follow-up visit. Depending on where the patient was being seen, the questionnaire was given out using a standardized script by either the radiation oncologist, radiation therapist, nurse, or volunteer. Laminated copies of the questionnaire and script were available at the CT simulator for each centre (e.g., in the event of a network connection loss or a patient who is unable to use a tablet). If the patient did not speak English, questionnaires could be completed with the assistance of an interpreter. Respondents had to have completed at least one survey in addition to the baseline survey to be included in the study. Our study was approved by the joint BC Cancer and University of British Columbia Research Ethics Board. 

Questionnaire responses were summarized with descriptive statistics. Given the long survey length, questions 14 and 26 were assessed as screening questions for any GI or GU toxicity (respectively) as they showed the highest event rates. Logistic regression was subsequently performed on these summary questions to associate the likelihood of a week 5 score being equal to 4 (“big problem”) given the week 2 score, while controlling for the baseline score. Clinical charts were abstracted to determine patient demographics, disease sites, treatment modality, and RT prescription/technique. Univariate comparisons of these potential confounders were performed using the chi-square statistic. All tests were 2-sided, with *p* < 0.05 considered statistically significant. SPSS statistical software package, version 21.0 (Chicago, IL, USA) was used. We did not analyze Supplementary Materials (Supplementary Figure S1) on General Health, as our main objective was to assess trends in acute radiation toxicity.

## 3. Results

A total of 1256 patients underwent a ≥25 fraction course of pelvic radiotherapy from March 2015 to May 2019, of which 698 completed a baseline and at least one follow-up questionnaire. The media age was 60 years (range 24–89). Of these patients, 50% and 33% had primary endometrial and cervical cancers, respectively. Approximately 58% were post-operative courses. Of the patients, 35% underwent concurrent chemotherapy, and 76% received between 45–50 Gy. Baseline patient and treatment characteristics are summarized in Table 1.

**Figure 1:**
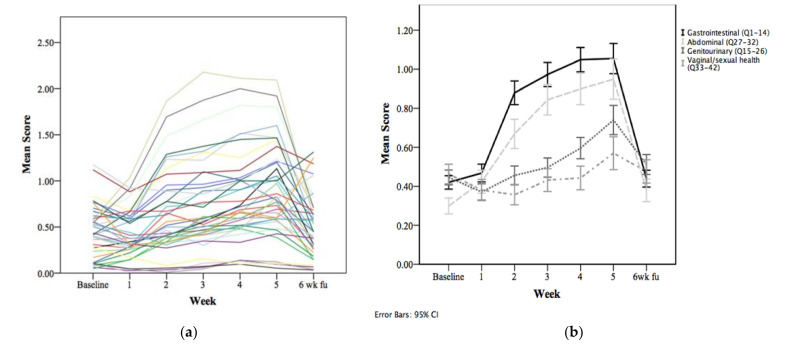
(**a**) Acute toxicity pattern of onset and resolution of symptoms is shown through mean question score, week by week. Each line represents a question. (**b**) This is confirmed from a systems perspective.

Mean question scores are shown in Table 2 and Figure 1a,b. Excluding questions on vaginal and sexual health, a pattern of onset and resolution of acute toxicity was detected in 27/32 questions. We defined this pattern as at least two successive increasing scores from baseline/week 1 during treatment, with improvement at the 6-week follow-up appointment compared to week 5. The exceptions were questions 15, 16, 18, 20, and 22—asking about urinary leakage and urinary bleeding. On the sub-analysis of mean scores based on primary tumor site, the questionnaire was similarly able to capture patterns of acute toxicity (endometrial 26/32, cervical 28/32, ovarian 29/32, vaginal 30/32, and vulvar 25/32 questions).

The sensitivity and specificity of the GI summary toxicity question was 69.2% and 93.2%. This was comparable to the GU summary toxicity question with 56.4% and 99.7%, respectively. If the definition of “no toxicity” for the GI summary question allowed a score of 0 or 1, the sensitivity increased further to 80.7% with a specificity of 73.7%. For the GU summary question, it was 78.8% and 83.4%, respectively. These results are summarized in Table 3.

A total of 695 patients were included in our predictive endpoint analysis. Of these, 384 responded to the GU summary question (question 26) both during weeks 2 and 5. Similarly, 377 responded to the GI summary question (question 14) during both those weeks. Only 6.3% of patients had a week 5 score of 4 (where toxicity is a “big problem”) for the GU summary question, and even less (1.9%) for the GI summary question. 

Logistic regression showed that an increase in week 2 scores from baseline lead to an increased risk of week 5 scores being equal to 4 (controlled for baseline score; GU summary, OR = 2.2, 95% CI 1.48–3.42, *p* < 0.001; GI summary, OR = 2.2, 95% CI 1.04–4.80, *p* < 0.05). The low number of events (week 5 scores = 4) for both summary questions did not allow for the univariate results to be productively added to a multivariate model, though primary tumor site was found to be significant (*p* = 0.01) for GI toxicity. The results of the univariate analyses are shown in Table 4 and Table 5.

**Figure 2:**
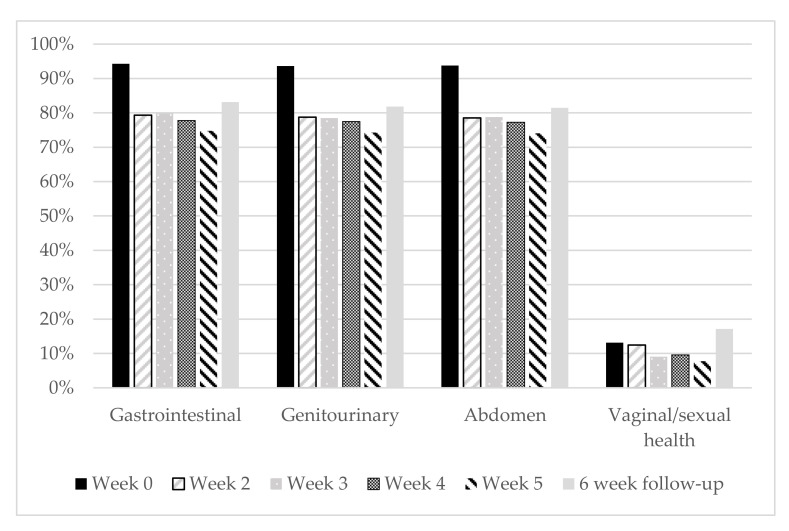
Domain response rates compared to baseline. Response was considered positive if ≥60 percent of the questions within that domain were answered.

Question response rates across all domains decreased by approximately 21% by week 5 with an 8.4% increase at the 6-week follow-up appointment, with the exception of the questions on sexual and vaginal health. On average, only 13% responded to these questions at each time point. These results are shown in Figure 2. 

## 4. Discussion

The Canadian Partnership for Quality Radiotherapy (CPQR) has strongly recommended utilizing PROs in clinical practice, with an emphasis on populations with typically high symptom burden such as patients with gynecologic cancers [5]. To our knowledge, this is one of the first studies to compare weekly PRO data on acute radiation toxicity in patients with gynecologic cancers. We were able to detect the onset and resolution of acute toxicity in patients undergoing pelvic radiotherapy. Exceptions to this were urinary incontinence (with 58% of patients being postoperative, 27% having baseline incontinence, and which persisted at 6-week follow-up), urinary bleeding (which improved throughout RT), and sexual and vaginal health toxicity (in which patients are generally not sexually active during treatment and toxicity is subacute).

Overall rates of GI and GU toxicity were comparable to clinician-reported outcomes in the literature [6,7]. For example, 65% reported worsening bowel symptoms at week 5 compared to baseline in the summary GI question compared to a 53.8% physician-reported grade 1–2 GI toxicity rate in the PORTEC-2 study. It is possible that our reported GI toxicity rates could be slightly lower as questionnaire response rates were approximately 20% less at week 5 compared to baseline, and well-patients could have been less likely to respond [8]. Previous studies have also shown the potential for physician under-reporting of toxicity, possibly due to the lack of reporting of side effects not felt directly attributable to radiotherapy [9,10]. This could be applicable to our cohort in which 58% initially underwent surgery and 35% concurrent chemotherapy. By week 5, we also found that 32% (164/505, question 3) of respondents noted diarrhea on at least a daily basis. This is supported by a recently published randomized trial by Klopp et al. comparing the toxicity of standard four-field radiotherapy to pelvic IMRT using PRO data, where frequent or constant diarrhea was observed in 51.9% and 33.7%, respectively [11].

Using logistic regression, we were able to demonstrate that PRO responses could predict for significant downstream GI or GU toxicity based on increased early toxicity scores. This could not be evaluated further on multivariate analysis due to a low number of significant toxic events and insufficient sample size. This is not particularly surprising though, as rates of severe toxicity reported in the literature are low, typically <10% grade ≥3 GI and <5% grade ≥3 GU CTCAE toxicity [12,13,14,15]. Primary tumor site was a significant variable in the GI univariate analysis and likely relates to the anatomical distribution of radiation dose in proximity to bowel. Similarly, a trend was observed for radiation technique (*p* = 0.06), though, interestingly, not for GI (which conformal techniques have commonly shown reduction in toxicity compared to 3D-CRT) but for GU toxicity [12,16]. It is possible this may at least be partly related to bladder blocks not being routinely used in 3D-CRT cases [17]. There was also a trend towards higher significant GI toxicity with concurrent chemotherapy (*p* = 0.06), which has been shown in previous studies [18]. Developing a predictive model for relevant toxicity levels could provide opportunities for early intervention (e.g., dietary counseling and information on anti-diarrheals in patients experiencing early toxicity that would predict “at least daily diarrhea” or diarrhea that would be a “big problem” by week 5), which could reduce treatment interruptions and improve locoregional control.

Our survey response rate was >70% at week 5 in eligible patients. While there is no scientifically proven minimum response rate, 60% has been used by some as a measure of survey quality [8]. It is notable that only 698 of the 1256 patients that underwent a 5-week course of radiotherapy during the study period completed a baseline plus additional questionnaire. Part of this discrepancy could have been attributable to a delay in cancer centres adopting the questionnaire into routine practice, and it is reassuring that a significant drop-off in response rate was not observed in the study patients who completed two or more questionnaires. Reasons why patients declined to answer our questionnaire were not documented, but we hypothesize the reasons are partly related to its 49-question length. This would be consistent with implementation issues (e.g., 20 question limit) previously described in the literature [19]. Despite wording that was not entirely open-ended, we found that questions 14 and 26 could potentially be used as summary GI and GU questions with acceptable sensitivity and specificity, if clinically meaningful toxicity (where one may intervene) was considered a score of ≥2. Given our findings, we would recommend screening questions be considered in a tree-format questionnaire to reduce completion times and improve uptake.

While there was no potential surrogate in our questionnaire, using a screening question to inquire about vaginal and sexual toxicity may not be effective. Multiple studies have identified complex physical and psychosocial patient concerns after treatment, and a screening question could serve as a barrier for a topic that patients have shown to be more hesitant to discuss with their healthcare providers [20,21,22]. This is consistent with our finding that patients were approximately 6 times less likely to respond to questions about vaginal and sexual health. 

Our study must be interpreted within the context of its strengths and limitations. Given the rarity of significant GI or GU toxicity events, our sample size was not large enough to build a strong predictive model for toxicity. The lack of true screening questions within our questionnaire limited our ability to assess whether a tree-format could be used over its 49-question length. The lack of vaginal and sexual health question responses also limited our ability to evaluate this important toxicity. Lastly, the questionnaire was able to follow acute toxicity patterns across different primary tumor sites, however, it may require some modification depending on the site, as not all side effects were captured (e.g., skin toxicity for vulvar cancers). Strengths of our study include the number of data time points to assess patterns, a good questionnaire response rate in eligible patients, and our population-based radiotherapy program, which routinely distributes PRO questionnaires during radiotherapy free from selection bias. 

By demonstrating that PRO data can be used to track acute toxicity during radiotherapy, future directions should move towards using this tool to provide more complex, adaptive care such as early side effect management to mitigate severe toxicity, potentially modifying radiation delivery while on treatment (e.g., magnetic resonance-guided RT), and for better evaluating how treatment modifications may affect patient outcomes on a broader level (e.g., dosimetric changes, brachytherapy, concurrent systemic therapy) [23,24,25].

## Figures and Tables

**Table 1:** Baseline characteristics.

Variable		(*n* = 698)
Age (years)	<50 50–69 ≥70	22% 55% 23%
Intent	Radical Post-op	42% 58%
Primary tumor	Endometrial Cervical Ovarian Vulvar Vaginal Unknown	50% 33% 9% 5% 3% 0.1%
Concurrent chemotherapy	No Yes	65% 35%
Brachytherapy	None Intracavitary Interstitial Vaginal Vault	59% 27% 0.7% 14%
Radiation Field	Local Only Whole Pelvis ± Boost	3% 97%
Total Dose (Gy)	<45 45–50 >50	0.1% 76% 24%
Whole Pelvic Field Dose (Gy)	<45 45 45.1–50	0.4% 95% 4%
Whole Pelvic Field Dose Per Fraction (Gy)	1.5–1.79 1.8–2	4% 96%
Technique	3D-CRT IMRT/VMAT	25% 75%

Abbreviations: *3D-CRT* three-dimensional conformal radiotherapy, *IMRT* intensity-modulated radiotherapy, *VMAT* volumetric modulated arc therapy.

**Table 2:** Mean questionnaire scores.

Question	Mean Score (Δ Compared to Baseline)						
	Baseline	Wk1	Wk2	Wk3	Wk4	Wk5	6 Wk Fu
Section 1–Bowel Functions							
1. How often have you had rectal urgency (felt like you had to pass stool, but did not) during the last 7 days?	0.68	0.71 (+4%)	0.74 (+9%)	0.97 (+43%)	0.92 (+35%)	0.94 (+38%)	0.49 (−28%)
2. How often have you had uncontrolled leakage of stool or feces during the last 7 days?	0.14	0.10 (−29%)	0.25 (+79%)	0.49 (+250%)	0.43 (+207%)	0.40 (+186%)	0.23 (+64%)
3. How often have you had stools (bowel movements) that were loose or liquid (no form, watery, mushy) during the last 7 days?	0.58	0.59 (+2%)	1.18 (+103%)	1.92 (+231%)	1.93 (+233%)	1.93 (+233%)	0.70 (+21%)
4. How often have you had bloody stools during the last 7 days?	0.07	0.04 (−43%)	0.05 (−29%)	0.08 (+14%)	0.13 (+86%)	0.17 (+143%)	0.08 (+14%)
5. How often have you had crampy pain in your abdomen, pelvis, or rectum during the last 7 days?	0.81	0.64 (−21%)	0.80 (−1%)	1.10 (+36%)	1.19 (+47%)	1.22 (+51%)	0.74 (−9%)
6. How many bowel movements have you had on a typical day during the last 7 days?	0.23	0.24 (+4%)	0.37 (+61%)	0.58 (+152%)	0.56 (+143%)	0.64 (+178%)	0.32 (+39%)
7. How often have your bowel movements been painful during the last 7 days?	0.39	0.36 (−8%)	0.37 (−5%)	0.64 (+64%)	0.81 (+108%)	0.87 (+123%)	0.46 (+18%)
How big a problem, if any, has each of the following been for you during the last 7 days?							
8. Urgency to have a bowel movement	0.58	0.54 (−7%)	0.82 (+41%)	1.29 (+122%)	1.22 (+110%)	1.30 (+124%)	0.76 (+31%)
9. Increased frequency of bowel movements	0.37	0.38 (+3%)	0.83 (+124%)	1.33 (+259%)	1.39 (+276%)	1.34 (+262%)	0.56 (+51%)
10. Watery bowel movements	0.42	0.38 (−10%)	0.78 (+86%)	1.40 (+233%)	1.33 (+217%)	1.34 (+219%)	0.54 (+29%)
11. Losing control of your stools	0.19	0.13 (−32%)	0.31 (+63%)	0.58 (+205%)	0.56 (+195%)	0.54 (+184%)	0.33 (+74%)
12. Bloody stools	0.10	0.07 (−30%)	0.06 (−40%)	0.07 (−30%)	0.11 (+10%)	0.15 (+50%)	0.10 (0%)
13. Abdominal/Pelvic/Rectal Pain	0.72	0.58(−19%)	0.66(−8%)	0.87 (+21%)	0.98 (+36%)	1.00 (+39%)	0.71 (−1%)
14. Overall, how big a problem have your bowel habits been for you during the last 7 days?	0.64	0.75 (+17%)	1.05 (+64%)	1.54 (+141%)	1.59 (+148%)	1.64 (+156%)	0.82 (+28%)
Section 2–Urinary Functions							
15. Over the past 7 days, how often have you leaked urine?	0.69	0.54 (−22%)	0.52 (−25%)	0.47 (−32%)	0.53 (−23%)	0.56 (−19%)	0.66 (−4%)
16. Over the past 7 days, how often have you urinated blood?	0.12	0.08 (−33%)	0.05 (−58%)	0.11 (−8%)	0.06 (−50%)	0.10 (−17%)	0.03 (−75%)
17. Over the past 7 days, how often have you had pain or burning with urination?	0.24	0.15 (−37%)	0.27 (+13%)	0.48 (+100%)	0.68 (+183%)	0.95 (+296%)	0.33 (+38%)
18. Which of the following best describes your urinary control during the last 7 days?	0.51	0.36 (−29%)	0.36 (−29%)	0.38 (−25%)	0.43 (−16%)	0.44 (−14%)	0.49 (−4%)
19. How many pads or adult diapers per day did you usually use to control leakage during the last 7 days?	0.39	0.37 (−5%)	0.39 (0%)	0.40 (+3%)	0.48 (+23%)	0.47 (+21%)	0.40 (+3%)
How big a problem, if any, has each of the following been for you during the last 7 days?							
20. Dripping or leaking urine	0.63	0.49 (−22%)	0.47 (−25%)	0.45 (−29%)	0.47 (−25%)	0.52 (−17%)	0.60 (−5%)
21. Pain or burning on urination	0.22	0.17 (−23%)	0.25 (+14%)	0.40 (+82%)	0.59 (+168%)	0.76 (+245%)	0.32 (+45%)
22. Bleeding with urination	0.14	0.09 (−36%)	0.05 (−64%)	0.11 (−21%)	0.07 (−50%)	0.09 (−36%)	0.04 (−71%)
23. Weak urine stream or incomplete emptying	0.43	0.34 (−21%)	0.33 (−23%)	0.40 (−7%)	0.46 (+7%)	0.51 (+19%)	0.36(−16%)
24. Waking up to urinate	1.19	0.98 (−18%)	1.08 (−9%)	1.13 (−5%)	1.15 (−3%)	1.31 (+10%)	1.20 (+1%)
25. Need to urinate frequently during the day	0.83	0.70 (−16%)	0.81 (−2%)	0.92 (+11%)	0.97 (+17%)	1.11 (+34%)	0.92 (+11%)
26. Overall, how big a problem have your urinary function been for you during the last 7 days?	0.72	0.55 (−24%)	0.68 (−6%)	0.72 (0%)	0.92 (+28%)	1.04 (+44%)	0.82 (+14%)
Section 3–Abdominal Problems							
27. In the last 7 days, what was the severity of your pain in the abdomen (belly area) at its worst?	0.64	0.56 (−12%)	0.62 (−3%)	0.73 (+14%)	0.85 (+33%)	0.89 (+39%)	0.61 (−5%)
28. In the last 7 days, how much did pain in the abdomen (belly area) interfere with your usual or daily activities?	0.45	0.39 (−13%)	0.40 (−11%)	0.50 (+11%)	0.55 (+22%)	0.64 (+42%)	0.43 (−4%)
29. In the last 7 days, how often did you have loose or watery stools (diarrhea)?	0.55	0.50 (−9%)	1.07 (+95%)	1.70 (+209%)	1.76 (+220%)	1.81 (+229%)	0.78 (+42%)
30. In the last 7 days, how often did you lose control of bowel movements?	0.14	0.13 (−7%)	0.25 (+79%)	0.48 (+243%)	0.50 (+257%)	0.45 (+221%)	0.26 (+86%)
31. In the last 7 days, how much did loss of control of bowel movements interfere with your usual or daily activities	0.14	0.14 (0%)	0.27 (+93%)	0.58 (+314%)	0.66 (+371%)	0.64 (+357%)	0.30 (+114%)
32. In the last 7 days, how often on average have you taken an anti-diarrhea medication?	0.06	0.07 (+17%)	0.16 (+167%)	0.49 (+717%)	0.70 (+1067)	0.74 (+1133)	0.16 (+167%)
Section 4–Gynecologic Problems							
33. Have you had irritation or soreness in your vagina or vulva during the past 4 weeks?	0.40	0.26 (−35%)	0.28 (−30%)	0.40 (0%)	0.52 (+30%)	0.59 (+48%)	0.44 (+10%)
34. Have you had discharge from your vagina during the past 4 weeks?	0.57	0.50 (−12%)	0.39 (−32%)	0.35 (−39%)	0.40 (−30%))	0.41 (−28%)	0.42 (−26%)
35. Have you had abnormal bleeding from your vagina during the past 4 weeks?	0.39	0.24 (−38%)	0.16 (−59%)	0.17 (−56%)	0.15 (−62%)	0.14 (−64%)	0.08 (−79%)
36. Have you felt dissatisfied with your body during the past 4 weeks?	0.68	0.61 (−10%)	0.61 (−10%)	0.71 (+4%)	0.74 (+9%)	0.78 (+15%)	0.69 (+1%)
37. Have you worried that sex would be painful during the past 4 weeks?	0.53	0.48 (−9%)	0.46 (−13%)	0.39 (−26%)	0.48 (−9%)	0.52 (−2%)	0.68 (+28%)
38. Have you been sexually active during the past 4 weeks?	0.18	0.25 (+39%)	0.23 (+28%)	0.18 (0%)	0.18 (0%)	0.16 (−11%)	0.29 (+61%)
39. Has your vagina felt dry during sexual activity during the past 4 weeks?	0.99	0.87 (−12%)	0.89 (−10%)	0.91 (−8%)	0.96 (−3%)	0.92 (−7%)	1.04 (+5%)
40. Has your vagina felt short during the past 4 weeks?	0.45	0.31 (−31%)	0.40 (−11%)	0.42 (−7%)	0.56 (+24%)	0.57 (+27%)	0.88 (+96%)
41. Has your vagina felt tight during the past 4 weeks?	0.53	0.63 (+19%)	0.66 (+25%)	0.84 (+58%)	0.77 (+45%)	0.79 (+49%)	1.10 (+108%)
42. Have you had pain during sexual intercourse or other sexual activity during the past 4 weeks?	0.65	0.48 (−26%)	0.34 (−48%)	0.43 (−34%)	0.54 (−17%)	0.67 (+3%)	0.95 (+46%)
43. Was sexual activity enjoyable for you during the past 4 weeks?	1.91	1.85 (−3%)	1.91 (0%)	1.82 (−5%)	1.87 (−2%)	1.79 (−6%)	1.40 (−27%)

Δ Change in score compared to baseline.

**Table 3:** Sensitivity and specificity of summary questions.

		Sub-Question Toxicity ^1^					
		Positive	Negative	Sensitivity	Specificity	PPV	NPV
GI summary question (#14) *	Positive	1957	33	69.2%	93.2%	98.3%	34.4%
	Negative	869	455				
GU summary question (#26) ^+^	Positive	1472	2	56.4%	99.7%	99.9%	36.8%
	Negative	1138	663				

^1^ GI sub-questions are #1–13 and GU sub-questions are #15–25; Abbreviations: PPV = positive predictive value; NPV = negative predictive value; * *Overall, how big a problem have your bowel habits been for you during the last 7 days?;* ^+^ *Overall, how big a problem have your urinary function been for you during the last 7 days?*

**Table 4:** Univariate analysis of increased week 2 GU toxicity leading to severe week 5 toxicity (using GU summary question).

Variable		Week 5 Score of 4 (Total Cases)	*p*-Value
Technique	4 Field IMRT VMAT	11.6% (112) 10.0% (20) 5.5% (363)	0.06
Primary Site	Cervix Endometrium Ovary Vagina Vulva Other	7.4% (163) 7.4% (242) 4.6% (44) 7.1% (14) 4.6% (22) 9.1% (11)	0.98
Concurrent Chemo	No Yes	6.6% (333) 8.0% (163)	0.58
Brachytherapy	No Yes	7.6% (303) 6.2% (193)	0.60
Total Dose	Mean (cGy)	4830.6 (4814.6 *)	0.88

* Mean dose of cases with a week 5 score less than 4.

**Table 5:** Univariate analysis of increased week 2 GI toxicity leading to severe week 5 toxicity (using GI summary question).

Variable		Week 5 Score of 4 (Total Cases)	*p*-Value
Technique	4 Field IMRT VMAT	4.5% (111) 0% (20) 2.0% (357)	0.28
Primary Site	Cervix Endometrium Ovary Vagina Vulva Other	10.1% (79) 4.5% (157) 0.8% (242) 0% (11) 15.4% (13) 0% (44)	0.01
Concurrent Chemo	No Yes	1.5% (331) 4.4% (158)	0.06
Brachytherapy	No Yes	2.7% (299) 2.1% (190)	0.77
Total Dose	Mean (cGy)	4923.3 (4810.4 *)	0.52

* Mean dose of cases with a week 5 score less than 4.

## Data Availability

The data presented in this study are available on request from the corresponding author.

## Supplementary Materials

The following supporting information can be downloaded at: https://www.mdpi.com/article/10.3390/curroncol29050270/s1, Figure S1: Patient-reported outcome questionnaire.
