# Supplementary material for: Using a Weekly Patient-Reported Outcome Questionnaire to Track Acute Toxicity in Patients Undergoing Pelvic Radiotherapy for Gynecologic Cancers
Source: Curr Oncol. 2022 May 5;29(5):3306–17. doi: 10.3390/curroncol29050270 (PMC9140006; doi:10.3390/curroncol29050270)
Supplement: Supplementary file 1 [file curroncol-29-00270-s001.zip › curroncol-1672008-supplementary.pdf]

Section 1 – Bowel Functions:

		Rarely or never	About once a week	More than once a week	About once a day	More than once a day
1	How often have you had rectal urgency (felt like you had to pass stool, but did not) during the last 7 days?	0	1	2	3	4
2	How often have you had uncontrolled leakage of stool or feces during the last 7 days?	0	1	2	3	4
3	How often have you had stools (bowel movements) that were loose or liquid (no form, watery, mushy) during the last 7 days?	0	1	2	3	4
4	How often have you had bloody stools during the last 7 days?	0	1	2	3	4
5	How often have you had crampy pain in your abdomen, pelvis or rectum during the last 7 days?	0	1	2	3	4

		Two or less	Three to four	Five or more
6	How many bowel movements have you had on a typical day during the last 7 days?	0	1	2

		Never	Rarely	About half the time	Usually	Always
7	How often have your bowel movements been painful during the last 7 days?	0	1	2	3	4

	**How big a problem, if any, has each of the following been for you during the last 7 days?**	No Problem	Very Small Problem	Small Problem	Moderate Problem	Big Problem
8	Urgency to have a bowel movement	0	1	2	3	4
9	Increased frequency of bowel movements	0	1	2	3	4
10	Watery bowel movements	0	1	2	3	4
11	Losing control of your stools	0	1	2	3	4
12	Bloody stools	0	1	2	3	4
13	Abdominal/Pelvic/Rectal Pain	0	1	2	3	4

		No Problem	Very Small Problem	Small Problem	Moderate Problem	Big Problem
14	Overall, how big a problem have your bowel habits been for you during the last 7 days?	0	1	2	3	4

Section 2 – Urinary Functions:

		Rarely or never	About once a week	More than once a week	About once a day	More than once a day
15	Over the past 7 days, how often have you leaked urine?	0	1	2	3	4
16	Over the past 7 days, how often have you urinated blood?	0	1	2	3	4
17	Over the past 7 days, how often have you had pain or burning with urination?	0	1	2	3	4

		Total control	Occasional dribbling	Frequent dribbling	No urinary control whatsoever
18	Which of the following best describes your urinary control during the last 7 days?	0	1	2	3

		None	1 pad per day	2 pads per day	3 or more pads per day
19	How many pads or adult diapers per day did you usually use to control leakage during the last 7 days?	0	1	2	3

	**How big a problem, if any, has each of the following been for you during the last 7 days?**	No Problem	Very Small Problem	Small Problem	Moderate Problem	Big Problem
20	Dripping or leaking urine	0	1	2	3	4
21	Pain or burning on urination	0	1	2	3	4
22	Bleeding with urination	0	1	2	3	4
23	Weak urine stream or incomplete emptying	0	1	2	3	4
24	Waking up to urinate	0	1	2	3	4
25	Need to urinate frequently during the day	0	1	2	3	4

		No Problem	Very Small Problem	Small Problem	Moderate Problem	Big Problem
26	Overall, how big a problem have your urinary function been for you during the last 7 days?	0	1	2	3	4

Section 3 – Abdominal Problems:

		None	Mild	Moderate	Severe	Very severe
27	In the last 7 days, what was the severity of your pain in the abdomen (belly area) at its worst?	0	1	2	3	4

		None at all	A little bit	Somewhat	Quite a bit	Very much
28	In the last 7 days, how much did pain in the abdomen (belly area) interfere with your usual or daily activities?	0	1	2	3	4

		Never	Rarely	Occasionally	Frequently	Almost constantly
29	In the last 7 days, how often did you have loose or watery stools (diarrhea)?	0	1	2	3	4
30	In the last 7 days, how often did you lose control of bowel movements?	0	1	2	3	4
31	In the last 7 days, how much did loss of control of bowel movements interfere with your usual or daily activities	0	1	2	3	4

		Never	0-1 times daily	2-3 times daily	4-6 times daily	More than 6 times daily
32	In the last 7 days, how often on average have you taken an anti-diarrhea medication?	0	1	2	3	4

Section 4 – Gynecological Problems:

		None at all	A little bit	Quite a bit	Very much
33	Have you had irritation or soreness in your vagina or vulva during the past 4 weeks?	0	1	2	3
34	Have you had discharge from your vagina during the past 4 weeks?	0	1	2	3
35	Have you had abnormal bleeding from your vagina during the past 4 weeks?	0	1	2	3
36	Have you felt dissatisfied with your body during the past 4 weeks?	0	1	2	3
37	Have you worried that sex would be painful during the past 4 weeks?	0	1	2	3
38	Have you been sexually active during the past 4 weeks?	0	1	2	3
39	Has your vagina felt dry during sexual activity during the past 4 weeks?	0	1	2	3
40	Has your vagina felt short during the past 4 weeks?	0	1	2	3
41	Has your vagina felt tight during the past 4 weeks?	0	1	2	3
42	Have you had pain during sexual intercourse or other sexual activity during the past 4 weeks?	0	1	2	3
43	Was sexual activity enjoyable for you during the past 4 weeks?	0	1	2	3

Section 5 – General Health:

44. We would like you to indicate how good or bad your own health is today, in your opinion. The best state you can imagine is marked by 100 and the worst state you can imagine is marked by 0...

Worst Best

0	0-10	10-20	20-30	30-40	40-50	50-60	60-70	70-80	80-90	90-100	100
0	1	2	3	4	5	6	7	8	9	10	11

Please indicate one statement in each group below which best describes your health today...

45	Mobility	0	I have no problems walking about
		1	I have some problems walking about
		2	I am confined to bed

46	Self Care	0	I have no problems with self-care
		1	I have some problems washing or dressing myself
		2	I am unable to wash or dress myself

47	Usual Activities	0	I have no problems with performing my usual activities
		1	I have some problems with performing my usual activities
		2	I am unable to perform my usual activities

48	Pain Discomfort	0	I have no pain or discomfort
		1	I have moderate pain or discomfort
		2	I have extreme pain or discomfort

49	Anxiety Depression	0	I am not anxious or depressed
		1	I am moderately anxious or depressed
		2	I am extremely anxious or depressed

Figure S1. Patient-reported outcome questionnaire.
